# Rendering Hard Waters Soft

**Published:** 1873-10

**Authors:** 


### Rendering Hard Waters Soft.

It is well known that water usually owes its hardness principally to the presence of the soluble bicarbonates of lime and magnesia, and that by the addition of caustic lime these salts are precipitated in the form of insoluble carbonates. Since the introduction of this method of rendering hard water soft, much difficuly has been experienced in ascertaining just how much lime to add in each case. If an excess of lime is added to water used for a steam boiler, the lime itself deposits when the water is concentrated, and augments the incrustation which it was intended to prevent.

From carefully conducted quantitative chemical analysis, it is possible to calculate very nearly the quantity of lime required. A simpler, quicker, and cheaper method is, however, a volumetric one, invented by John Stingel, of Vienna. A saturated solution of pure quick-lime in distilled water is first made and allowed to settle. It is then decanted, and the quantity of lime in solution is determined by tiration, as follows: 10 cubic centimeters of nitric acid, of one tenth the normal strength, is colored red by a few drops of tincture of litmus. Into this the lime water is allowed to drop slowly from a graduated pipette, until the litmus indicates that the solution is neutral. Since 54 parts of nitric acid will be neutralized by 28 parts of oxide of calcium, or pure quick-lime, it is only necessary to divide 28 by the quantity of lime solution employed in neutralizing 54 parts of acid, to find how much pure lime was contained in one cubic centimeter of the solution.

Some of this saturated solution of lime is now allowed to drop slowly from the pipette into 100 c. c. of the water to be operated upon, stirring the latter continually. At first it remains clear, owing to the slight solubility of the carbonates of lime and magnesia, and when at length it gets cloudy, the precipitate dissolves on stirring. This point also having been passed, the precipitate finally refuses to dissolve, and the liquid remains milky. The addition of lime solution is to be continued as long as it increases the turbidity. If flakes of carbonate of lime and magnesia separate, it contains too much lime already, which is proved by taking out a drop on to a piece of tumeric-paper, or if much excess is present nitrate of silver will also show it.

Having ascertained how much of the test solution is required for 100 c. c. of the water to be used, and knowing also the amount of lime in that quantity of the test solution, it is easy to calculate how much lime must be added to soften any given quantity of the hard water. The lime actually used not being perfectly pure, it will of course be necessary to determine the percentage of insoluble matter in it, and also ascertain how much of the water used will be required to dissolve the requisite amount of lime.—*Boston Journal of Chemistry.*

